# Co-Processed Starch–Beeswax Composites as Natural Tablet Lubricants: Preparation, Characterization, and Performance Evaluation

**DOI:** 10.3390/pharmaceutics18080925

**Published:** 2026-07-28

**Authors:** Ornanong S. Kittipongpatana, Karnkamol Trisopon, Rewat Phongphisutthinant, Supakit Chaipoot, Nisit Kittipongpatana

**Affiliations:** 1 Department of Pharmaceutical Sciences, Faculty of Pharmacy, Chiang Mai University, Chiang Mai 50200, Thailand; ornanong.kit@cmu.ac.th (O.S.K.); karnkamol.t@cmu.ac.th (K.T.); 2 Multidisciplinary Research Institute, Chiang Mai University, Chiang Mai 50200, Thailand; rewat.p@cmu.ac.th (R.P.); supakit.ch@cmu.ac.th (S.C.)

**Keywords:** lubricant, starch, beeswax, composite, pharmaceutical excipient, direct compression

## Abstract

**Background:** The development of naturally derived pharmaceutical excipients has attracted increasing interest as alternatives to conventional synthetic materials. **Methods:** In this study, starch–beeswax composites were prepared using native rice starch (RS) and spray-dried rice starch (SDRS) through melt levigation (ML) and emulsification (EM) techniques at starch-to-beeswax ratios of 9:1, 8:2, and 7:3. The physicochemical properties, surface hydrophobicity, morphology, tabletability, and lubrication performance of the resulting composites were evaluated and compared with magnesium stearate (MGS) and hydrogenated vegetable oil (HVO). **Results:** Co-processing with beeswax markedly increased the water contact angle from 35.4° and 59.7° for RS and SDRS, respectively, to values ranging from 94.5° to 125.1°, indicating successful modification of surface hydrophobicity. SEM analysis demonstrated changes in particle morphology and surface appearance following co-processing, while FT-IR confirmed the coexistence of characteristic starch- and beeswax-associated spectral features without evidence of detectable covalent modification. Co-processed formulations generally maintained or improved tabletability relative to their corresponding starch bases, with SDRS-based composites producing substantially harder tablets than RS-based formulations. The composites also reduced tablet ejection force and improved tablet mechanical properties compared with lubricant-free formulations. Among all samples, SDRS-EM-73 exhibited the best overall performance, reducing ejection force from 386.5 N for the lubricant-free control to 89.2 N, a value comparable to HVO (93.0 N), while producing tablets with high hardness (60.9 N), low friability (0.16%), and acceptable disintegration time (44.8 s). **Conclusions:** These findings demonstrate that co-processed starch–beeswax composites, particularly SDRS-EM-73, show considerable potential as naturally derived excipients for tablet manufacturing and may serve as sustainable alternatives to conventional tablet lubricants.

## 1. Introduction

The manufacture of pharmaceutical tablets requires careful control of friction throughout powder handling and compression. During compaction, friction between powder particles, die walls, and punch surfaces can adversely affect powder flow, tablet ejection, mechanical integrity, and production efficiency [1,2]. Lubrication is therefore essential for successful tablet manufacture because it reduces die-wall friction, minimizes sticking, improves powder flow, and facilitates tablet ejection [1]. Conventional pharmaceutical lubricants are typically hydrophobic materials that reduce friction between the tablet and die wall during compression and facilitate tablet ejection [2,3]. Examples include metallic stearates, particularly magnesium stearate, fatty acids such as stearic acid, sodium stearyl fumarate, and hydrogenated vegetable oils. These materials differ in lubrication efficiency and in their effects on powder compaction and tablet properties. Among these lubricants, magnesium stearate remains the most widely used because of its high lubrication efficiency at relatively low concentrations [3]. However, its hydrophobic surface coverage can, depending on formulation composition and processing conditions, interfere with interparticulate bonding and water penetration. Excessive concentration or prolonged blending may therefore reduce tablet mechanical strength and, in susceptible formulations, lead to prolonged disintegration and slower drug dissolution [3,4,5]. These limitations have encouraged the development of alternative lubricant systems that can provide adequate lubrication while minimizing negative effects on tablet quality [6].

In addition to the limitations of conventional lubricants, growing interest in sustainable and renewable pharmaceutical excipients has increased the demand for naturally derived alternatives. In recent years, naturally derived excipients have attracted considerable attention because of sustainability concerns, environmental awareness, and the demand for renewable materials [7]. Consequently, biodegradable and biocompatible materials are being investigated as alternative tablet lubricants to reduce environmental impact while maintaining tablet quality [8]. Natural biopolymers and lipid-based materials are particularly attractive because of their biodegradability, biocompatibility, and wide availability [9]. Previous studies have investigated several naturally sourced materials, including rice bran derivatives, rice bran wax, and chitosan-based excipients for pharmaceutical applications and potential lubricant use [10,11]. For example, stabilized defatted rice bran and rice bran wax were shown to reduce tablet ejection force while maintaining acceptable tablet quality, demonstrating the potential of rice-derived materials as alternative lubricants [10]. However, these studies focused mainly on rice bran–based materials. Comparatively little attention has been given to co-processed starch–lipid systems designed specifically for tablet lubrication.

Starch is one of the most widely used pharmaceutical excipients because of its availability, low cost, low toxicity, and versatility [12]. It can be modified through physicochemical treatment and co-processing to obtain properties suitable for different pharmaceutical applications [12,13]. Among the various starch sources, rice starch is particularly attractive because of its small granule size, wide availability, and established use in pharmaceutical formulations. In addition, a commercially available spray-dried rice starch (SDRS; Era-Tab^®^) has been developed as a direct-compression rice starch excipient and has demonstrated favorable powder flow and tableting properties [10]. The availability of both native rice starch and SDRS provides an opportunity to compare the influence of starch processing on the performance of co-processed lubricant systems. Although starch itself is not an effective tablet lubricant, its versatility and ability to undergo physical modification and co-processing make it a potential carrier matrix for functional hydrophobic components. Therefore, incorporating hydrophobic components into starch matrices may provide a useful approach for improving lubrication performance.

Beeswax is a natural wax composed mainly of long-chain esters, hydrocarbons, free fatty acids, and fatty alcohols. Its hydrophobicity, biocompatibility, low toxicity, and established pharmaceutical use as a matrix-forming, viscosity-modifying, and release-controlling excipient make it a promising candidate for lubricant development [14,15]. In addition, its thermoplastic nature and relatively low melting range allow it to be incorporated into powder systems through melt-based processing methods [14]. The incorporation of lipidic materials into carbohydrate-based matrices has been explored as a strategy to modify material properties and improve functional performance. For example, beeswax-functionalized starch–cellulose composites showed reduced moisture absorption while maintaining mechanical strength, demonstrating the ability of lipidic materials to modify carbohydrate matrices [16]. More broadly, starch–lipid systems have been investigated in films, coatings, packaging materials, and controlled-release applications [17,18]. These studies suggest that lipid incorporation can modify surface properties and particle interactions, which may influence lubrication behavior in pharmaceutical powders.

Co-processing is an established excipient-engineering approach that combines two or more materials through physical processing to achieve improved functionality while maintaining the chemical identity of the individual components [19]. Compared with chemical modification or molecular complexation, co-processing provides a practical way to tailor material properties without introducing new chemical entities. Despite growing interest in starch–lipid materials, few studies have investigated co-processed rice starch–lipid systems specifically for pharmaceutical lubrication. In particular, the effects of composite composition and co-processing method on lubrication efficiency and tablet quality attributes have not been thoroughly investigated. A better understanding of these relationships may support the development of naturally derived lubricant alternatives for solid dosage formulations.

Therefore, this study aimed to develop rice starch–beeswax composites using heat-melt and emulsion-based co-processing methods and evaluate their potential as naturally derived tablet lubricants. Native rice starch (RS) and spray-dried rice starch (SDRS; Era-Tab^®^) were investigated as alternative starch bases for composite preparation. The physicochemical characteristics, surface properties, powder performance, lubrication efficiency, and effects on tablet quality attributes were systematically evaluated and compared with conventional lubricants, namely magnesium stearate and hydrogenated vegetable oil. The findings are expected to improve understanding of how co-processing methods and composition affect the lubrication performance of rice starch–beeswax systems and support the development of sustainable lubricants for solid dosage forms.

## 2. Materials and Methods

### 2.1. Materials

Beeswax was obtained from a local longan honey farm in Mae Wang District, Chiang Mai, Thailand. The material was not supplied as a certified pharmacopoeial-grade excipient and was physically purified before use, as described in Section 2.2. Rice starch (RS) was supplied by Thai Flour Industry Co., Ltd. (Bangkok, Thailand). The material was a purified and processed native rice starch, rather than rice flour, and was listed by the supplier for pharmaceutical applications; however, compliance of the specific batch with a named pharmacopoeial monograph was not independently verified. Spray-dried rice starch (SDRS, Era-Tab^®^) was supplied by Erawan Pharmaceutical Research and Laboratory (Bangkok, Thailand). Magnesium stearate was purchased from Union Science Chemical (Chiang Mai, Thailand). Hydrogenated cottonseed oil (HVO, Lubritab^®^) was obtained from JRS Pharma (Rosenberg, Germany). Polysorbate 80 (Tween 80) was from Sigma-Aldrich (St. Louis, MO, USA). All solvents used in this study were of analytical grade.

### 2.2. Beeswax Processing

Beeswax obtained from the local longan honey farm was supplied as partially cleaned chunks free of visible comb fragments and debris. The wax was further purified by a physical purification process without chemical treatment. Briefly, the beeswax was washed with distilled water, melted in a water bath at 65–75 °C, and filtered through muslin cloth to remove residual impurities. The filtered wax was then cooled slowly to allow sedimentation, and the purified upper layer was collected after solidification. The purified beeswax was characterized for its physicochemical properties, including melting point, saponification value, acid value, iodine value, and ester value, using methods previously described by Kittipongpatana et al. [10]. The purified beeswax exhibited a melting point of 62.5 °C, a saponification value of 101 mg KOH/g, an acid value of 20 mg KOH/g, an iodine value of 10.8 g I_2_/100 g, and an ester value of 81 mg KOH/g. The measured physicochemical values were consistent with reported ranges for beeswax used in pharmaceutical applications [14]; however, these results should not be interpreted as confirmation of compliance with all requirements of a specific pharmacopoeial monograph.

### 2.3. Preparation of Starch-Beeswax Composite

#### 2.3.1. Melt Levigation (ML)

Beeswax was melted in a mortar at approximately 60–65 °C, after which starch powder (RS or SDRS) was incorporated and mixed by levigation at starch-to-beeswax ratios of 9:1, 8:2, and 7:3), corresponding to beeswax contents of 10%, 20%, and 30% *w*/*w*, respectively. These ratios were selected to evaluate the effect of progressively increasing beeswax incorporation on the physicochemical, tableting, and lubrication properties of the co-processed systems while maintaining starch as the major matrix component. The temperature was maintained above 65 °C throughout the mixing process to maintain the beeswax in the molten state throughout mixing. The resulting composite was allowed to cool to room temperature and subsequently passed through sieve No. 60 prior to further evaluation.

#### 2.3.2. Emulsifying Beeswax (EM)

Beeswax was prepared as an oil-in-water (*o*/*w*) emulsion using Tween 80 as the emulsifying agent. Separate emulsions with a final mass of 30.0 g were prepared for the starch: beeswax ratios of 9:1, 8:2, and 7:3 described in Section 2.3.1. The beeswax: Tween 80 ratio was maintained at 5:1 *w*/*w*. Accordingly, the emulsions contained 2.0, 4.0, or 6.0 g beeswax; 0.4, 0.8, or 1.2 g Tween 80; and 27.6, 25.2, or 22.8 g purified water, respectively. Beeswax and Tween 80 were heated together at 75 °C with gentle stirring until the beeswax was completely melted and a homogeneous phase was obtained. Purified water was heated separately to the same temperature and gradually added to the molten phase under continuous homogenization at 8000 rpm for 5 min. The emulsions were then stirred continuously while cooling to room temperature. Any water lost through evaporation was replaced with purified water to restore the final mass to 30.0 g. Each complete emulsion was thoroughly mixed in a mortar with 18.0, 16.0, or 14.0 g starch for the 9:1, 8:2, and 7:3 formulations, respectively, until a homogeneous composite was obtained. The composites were dried at 50 °C for 24 h and passed through an ASTM E11 No. 60 test sieve with a nominal aperture of 250 µm before further evaluation.

### 2.4. Physicochemical Properties of Composite Rice Starch-Beeswax Sample

#### 2.4.1. Moisture Content

Moisture content was determined using an Ohaus MB25 moisture analyzer (Ohaus Corp., Parsippany, NJ, USA) equipped with a halogen heating unit. Samples were heated at 55 °C to prevent beeswax melting and dried to constant mass. Moisture content was expressed as percentage weight loss.

#### 2.4.2. Powder Flow

The flow properties of the co-processed samples were evaluated by determining the compressibility index (CI) and Hausner ratio (HR) [20]. Both parameters were calculated from the bulk and tapped densities of the powder samples. Briefly, 50 mL of powder was transferred into a 100 mL graduated cylinder and the sample weight was recorded. Bulk density and tapped density were calculated using Equations (1) and (2), respectively, as the ratio of powder weight to powder volume before tapping and after tapping. Tapping was performed using a jolting volumeter (Stampfvolumeter SVM, Erweka, Langen, Germany) until no further change in powder volume was observed. The compressibility index was calculated as the percentage difference between tapped density and bulk density relative to tapped density (Equation (3)), whereas the Hausner ratio was determined from the ratio of tapped density to bulk density (Equation (4)).

ρ_bulk_ = m/V_bulk_ (1)

ρ_tapped_ = m/V_tapped_ (2)

CI = 100 × (ρ_tapped_ − ρ_bulk_)/ρ_tapped_ (3)

HR = ρ_tapped_/ρ_bulk_ (4)

#### 2.4.3. Particle Size Analysis

Particle size was determined by laser diffraction using a Malvern Mastersizer S (Malvern Instruments, Malvern, UK). Samples were analyzed using wet dispersion in methanol (refractive index 1.33), with starch assigned a refractive index of 1.53. Each sample was measured in triplicate. The volume-weighted mean diameter, D[4,3], was reported as the average particle size.

#### 2.4.4. Contact Angle Measurement

Contact angle measurements were performed using a Theta Flow optical contact angle meter (Biolin Scientific, Espoo, Finland) equipped with OneAttension software (Version 4.2.1) via the sessile drop method. Powder samples were compacted in cylindrical molds (2.5 cm diameter × 1.0 cm height) to obtain a smooth surface. A 5 µL droplet of deionized water was deposited onto the sample surface, and the contact angle was recorded after 10 s using image analysis software. Measurements were taken at three different positions on each sample, and the experiment was repeated in triplicate. Results are expressed as mean values.

### 2.5. Scanning Electron Microscopic (SEM) Analysis

Scanning electron microscopy (SEM) was performed to examine the surface morphology, shape, and size of the granules using a TESCAN CLARA microscope (TESCAN, Brno, Czech Republic). The analysis was carried out at an acceleration voltage of 10 keV. Samples were mounted onto copper stubs using double-sided adhesive tape and subsequently sputter-coated with gold under vacuum using a Safematic CCU-010 coating system (Safematic GmbH, Zizers, Switzerland). SEM micrographs were captured at a magnification of 4000×.

### 2.6. Fourier Transform Infrared (FT-IR) Spectroscopy

FT-IR spectra were obtained using an Invenio S spectrophotometer (Bruker, Billerica, MA, USA). Prior to analysis, samples were dried at 60 °C for 24 h and finely pulverized with KBr. Spectral measurements were conducted in attenuated total reflectance (ATR) mode to evaluate potential chemical modifications and functional group changes associated with the beeswax incorporation.

### 2.7. Tabletability of Co-Process Powders and Tablet Compositions

Powder samples (250 mg) were compressed using a hydraulic press (Carver Inc., Wabash, IN, USA) fitted with 14.23 mm flat-faced punches at compression forces of 0.5, 1.0, and 2.0 ton. No lubricant was applied to the punch and die surfaces during compression. The hardness of the resulting compacts was determined in triplicate using a hardness tester (Erweka, Langen, Germany). Tabletability profiles were constructed by plotting compression pressure (MPa) against tablet hardness (N) for each sample.

### 2.8. Compression Test and Tablet Evaluation

Tablet formulations consisted of spray-dried rice starch (SDRS, Era-Tab^®^) as the diluent and either standard or test lubricants at concentrations ranging from 0.5 to 2.0% *w*/*w*. A control formulation containing SDRS without lubricant was also prepared. SDRS powder was passed through an ASTM E11 sieve No. 20 (nomimal aperture of 850 µm), whereas lubricants were passed through sieve No. 40 (nominal aperture of 425 µm) prior to blending. The powders were mixed using a UECM-RD cube mixer (Unity Equipment, Nonthaburi, Thailand) for 5 min. Tablet compression was performed using a Fette 102i Laboratory Tablet Press (Fette Compacting GmbH, Schwarzenbek, Germany) equipped with a three-chamber Fill-O-Matic feed frame and 8 mm round concave punches. The turret speed was maintained at 13 rpm. Pre-compression and main compression forces were set at 0.4 and 4.5 kN, respectively. Tablets were prepared at a batch size of 1 kg, a target weight of 300 mg, corresponding to approximately 3300 tablets, with a filling depth of 10.13 mm. The feed frame paddle speed was maintained at 10 rpm. Process parameters, including actual pre-compression force, main compression force, and ejection force, were recorded after 10 min of continuous operation. Tablet appearance was visually evaluated, while surface morphology and edge integrity were further examined using a stereomicroscope. The finished tablets were evaluated for weight variation, hardness, friability, and disintegration time according to USP procedures.

### 2.9. Statistical Analysis

Unless otherwise stated, experiments were performed in triplicate, and results are expressed as mean ± standard deviation. Statistical analysis was conducted using one-way analysis of variance (ANOVA) for comparisons among multiple groups. Significance tests of means were analyzed using Tukey’s honestly significant difference (HSD) multiple range test at a 95% confidence level (*p* < 0.05).

## 3. Results and Discussion

### 3.1. Preparation of Starch-Beeswax Composites

A total of 12 starch–beeswax composites were prepared from two starch materials, namely native rice starch (RS) and spray-dried rice starch (SDRS), using three starch-to-beeswax ratios (9:1, 8:2, and 7:3) and two preparation methods, melt levigation (ML) and emulsification (EM).

### 3.2. Physicochemical Properties of Composites

#### 3.2.1. Moisture Content

The moisture content of the starch-beeswax composite samples ranged from 9.37 ± 0.38% to 11.72 ± 0.66%. Native rice starch (RS) showed a moisture content of 10.23 ± 0.15%, whereas spray-dried rice starch (SDRS) showed a lower value of 8.97 ± 0.38% (Table 1). Overall, SDRS-based composites exhibited lower moisture than RS-based samples. This may be attributed to the physical characteristics of the starting material [20,21]. However, because specific surface area and water-sorption behavior were not directly evaluated in the present study, the mechanism underlying the observed difference in moisture content cannot be confirmed. The preparation method also affected moisture content. Composites prepared by emulsification showed slightly higher moisture contents than those prepared by melt levigation. The slightly higher moisture contents of some emulsion-prepared composites may reflect differences arising from the aqueous preparation and drying processes; however, the mechanism was not directly investigated. In contrast, melt levigation is a solvent-free process that does not involve an aqueous phase and is therefore less likely to contribute additional residual moisture. No consistent relationship was observed between the beeswax ratio and moisture content. Although increasing beeswax content could theoretically reduce water uptake due to the hydrophobic nature and moisture-barrier properties of beeswax [15,16,18], the observed values fluctuated among formulations. This suggests that moisture content was influenced more strongly by the starch base and preparation method than by beeswax ratio alone.

#### 3.2.2. Powder Flow

Carr’s index (CI) and Hausner ratio (HR) were determined to evaluate the flowability and packing characteristics of the co-processed powders. The CI values ranged from 15.9 ± 0.6% to 27.0 ± 0.5%, with corresponding HR values of 1.19 ± 0.01 to 1.37 ± 0.01 (Table 1). Native rice starch exhibited a CI of 23.5 ± 1.3% and an HR of 1.31 ± 0.02, whereas spray-dried rice starch (SDRS) showed markedly lower values (CI = 11.6 ± 1.4%, HR = 1.13 ± 0.02), indicating superior flowability. These findings are consistent with previous reports describing spray-dried rice starch (Era-Tab^®^) as a directly compressible excipient with favorable flow characteristics, as evidenced by its low percentage compressibility and good flowability [22]. Consistent with this trend, SDRS-based co-processed excipients generally exhibited lower CI and HR values than the corresponding rice starch formulations, particularly at beeswax ratios of 9:1 and 8:2, suggesting that the favorable flow properties of SDRS were largely retained after co-processing. The improved flowability of SDRS-based formulations is likely related to the more spherical morphology and smoother particle surfaces generated during spray drying, which reduce interparticle friction and enhance particle mobility compared with native rice starch granules [21,23]. Beeswax ratio also affected powder flow. Formulations containing 30% beeswax (7:3 ratio) generally exhibited higher CI and HR values than those containing 10% or 20% beeswax, indicating increased cohesiveness and reduced flowability. The preparation method had a smaller influence than starch type and beeswax ratio; however, melt-levigated SDRS formulations generally showed lower CI and HR values than the corresponding emulsified products. Overall, the co-processed excipients maintained acceptable flow properties, which may facilitate powder handling and blending during tablet manufacturing.

#### 3.2.3. Particle Size

The volume-weighted mean particle diameters, D[4,3], of RS, SDRS, and SDRS-EM-73 were 48.46 ± 0.17, 132.48 ± 8.20, and 164.73 ± 6.84 µm, respectively. SDRS showed a larger mean particle diameter than RS, consistent with its agglomerated spray-dried morphology. The higher D[4,3] value of SDRS-EM-73 may reflect further particle enlargement or agglomeration following emulsion-based co-processing.

#### 3.2.4. Contact Angle

**Figure 1:**
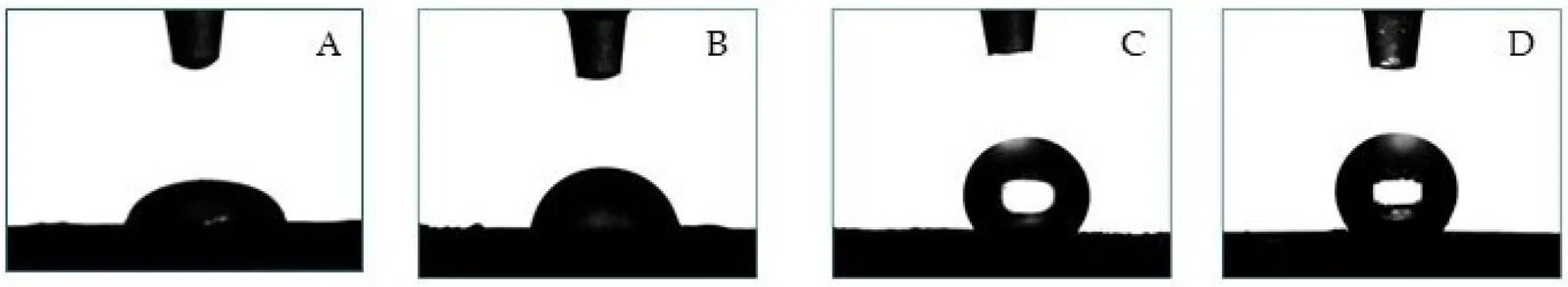
Water contact angle images of (**A**) native rice starch (RS), (**B**) spray-dried rice starch (SDRS), (**C**) native rice starch–beeswax composite prepared using the emulsion method (RS-EM), and (**D**) spray-dried rice starch–beeswax composite prepared using the emulsion method (SDRS-EM).

Water contact angle measurements were used to evaluate the apparent wettability of the compacted powder surfaces of the co-processed excipients (Table 1, Figure 1). Native rice starch and spray-dried rice starch (SDRS) exhibited contact angles of 35.4 ± 2.0° and 59.7 ± 2.7°, respectively, reflecting the hydrophilic nature of starch-based materials due to the abundance of surface hydroxyl groups. In contrast, MGS and HVO exhibited substantially higher contact angles of 133.6 ± 3.2° and 112.5 ± 5.3°, respectively, consistent with their highly hydrophobic character [6]. Co-processing with beeswax markedly increased the contact angle of both starch bases, yielding values between 94.5 ± 1.5° and 125.1 ± 7.9°. All co-processed formulations exhibited contact angles greater than 90°, indicating successful modification of the particle surface toward a hydrophobic character. The beeswax ratio had the greatest influence on surface hydrophobicity. For both RS- and SDRS-based formulations, increasing the beeswax content from 10% (9:1 ratio) to 30% (7:3 ratio) resulted in progressively higher contact angles, consistent with previous reports of increased surface water repellency following beeswax incorporation into starch-based and related hydrophilic matrices [16,24]. This trend is attributed to the hydrophobic long-chain hydrocarbons and esters present in beeswax, which reduce surface wettability and limit water penetration [15,25]. At equivalent beeswax ratios, the effects of starch type and preparation method were comparatively small. Although some SDRS formulations exhibited slightly higher contact angles than the corresponding RS formulations, this trend was not consistent across all samples. Similarly, emulsified products generally showed slightly higher contact angles than melt-levigated products, particularly at the 7:3 ratio. However, contact-angle measurements do not establish whether this difference resulted from a more uniform distribution of beeswax. Overall, apparent surface wettability was influenced primarily by beeswax content rather than consistently by starch type or preparation method.

The highest contact angles were observed for the 7:3 formulations, with SDRS-EM-73 (125.1 ± 7.9°), RS-EM-73 (123.0 ± 1.6°), and SDRS-ML-73 (122.8 ± 5.7°), exhibiting values approaching those of HVO and, to a lesser extent, MGS. These findings demonstrate that incorporation of beeswax effectively imparted hydrophobic surface characteristics comparable to those of conventional hydrophobic pharmaceutical excipients.

The 7:3 formulations also generally exhibited the lowest ejection forces within their respective series. The parallel trends between beeswax content, apparent surface wettability, and ejection force are consistent with the possibility that greater surface expression or availability of the hydrophobic wax phase contributed to reduced die-wall friction and improved lubrication performance [6]. However, this proposed relationship is based on indirect and complementary observations rather than direct evidence of a low-shear interfacial wax film. Contact-angle measurements may also be influenced by surface roughness, porosity, compaction characteristics, and liquid penetration and therefore do not determine the spatial distribution of beeswax or demonstrate the formation of a homogeneous or continuous surface coating.

### 3.3. Scanning Electron Micrographs (SEM)

**Figure 2:**
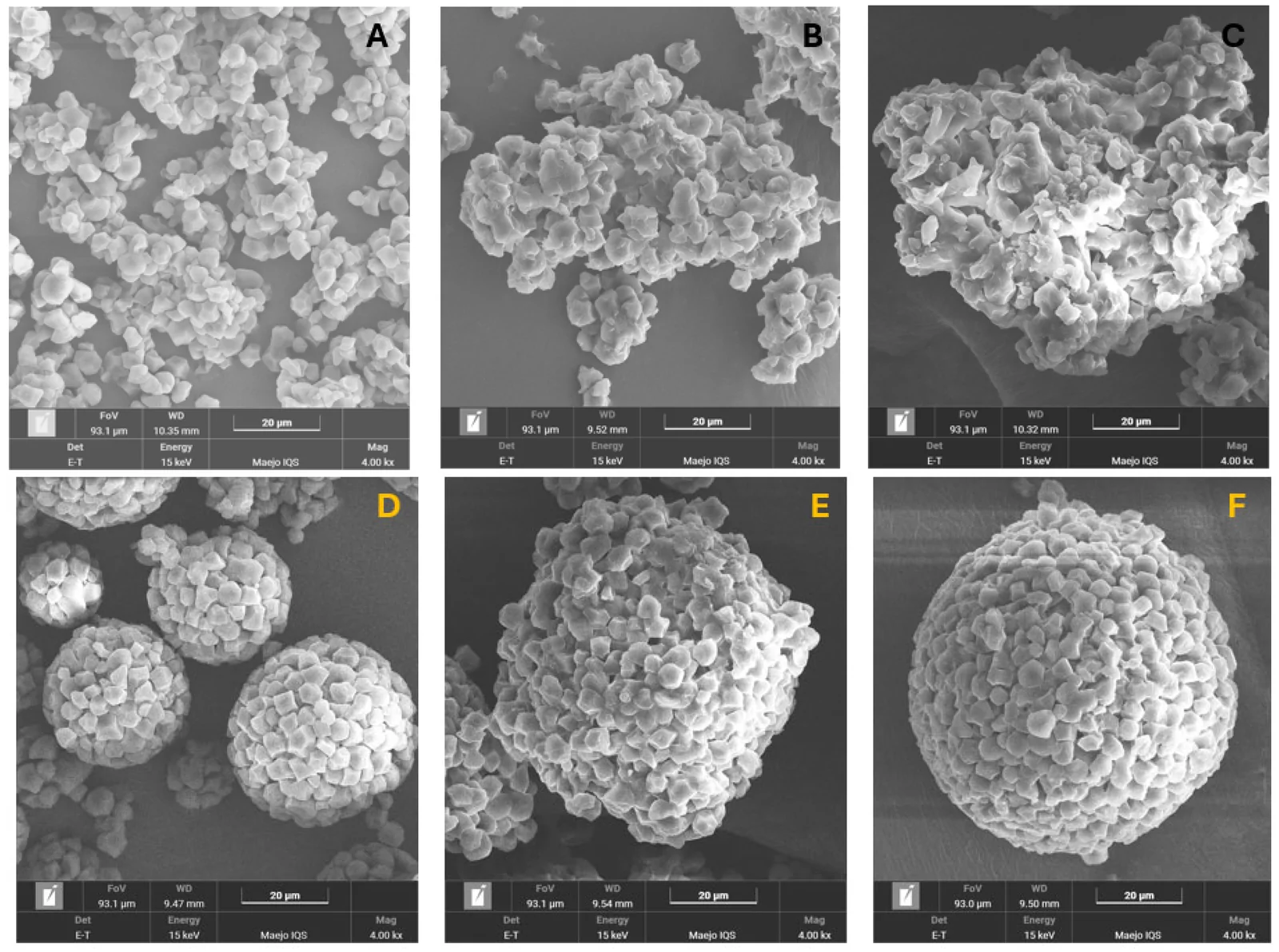
Scanning electron microscopy (SEM) images of (**A**) native rice starch (RS), (**B**) native rice starch–beeswax composite prepared by melt levigation (RS-ML), (**C**) native rice starch–beeswax composite prepared using the emulsion method (RS-EM), (**D**) spray-dried rice starch (SDRS), (**E**) spray-dried rice starch–beeswax composite prepared by melt levigation (SDRS-ML), and (**F**) spray-dried rice starch–beeswax composite prepared using the emulsion method (SDRS-EM), recorded at 4000× magnification. Scale bars = 20 µm.

Scanning electron microscopy (SEM) was used to examine the morphology of native rice starch (RS), spray-dried rice starch (SDRS), and their co-processed composites with beeswax (Figure 2). RS (Figure 2A) consisted of irregularly shaped granules that formed loosely packed aggregates. In contrast, SDRS particles (Figure 2D) exhibited a predominantly spherical morphology with relatively uniform particle size and a compact structure, characteristic of the original spray-dried material [21,26].

Following co-processing with beeswax, both RS- and SDRS-based formulations exhibited changes in particle aggregation and morphology. The RS-based composites, RS-ML and RS-EM (Figure 2B,C), appeared as larger and more compact agglomerates than the parent RS particles, with rougher and more irregular surfaces. In contrast, the SDRS-based composites, SDRS-ML and SDRS-EM (Figure 2E,F), largely retained the spherical architecture of the parent SDRS particles, although their surfaces became rougher and exhibited additional plate-like or particulate features.

Among the SDRS-based composites, SDRS-EM appeared to retain a more regular spherical morphology than SDRS-ML. No large, clearly separated wax-rich domains were visually apparent in the selected SEM fields. However, SEM provides qualitative information on particle morphology and surface appearance and does not directly determine the spatial distribution, continuity, or thickness of the beeswax phase within or on the composite particles. The observed changes should therefore be interpreted as evidence that co-processing altered particle aggregation and surface morphology rather than as direct confirmation of homogeneous beeswax distribution or the formation of a continuous surface coating.

Similar relationships among wax distribution, surface roughness, and hydrophobicity have been reported in related starch–wax and beeswax-containing systems, although their compositions and processing conditions differed from those used in the present study. Muscat et al. used chemically selective S-FTIR mapping to show that wax distribution within starch films was associated with surface roughness and contact angle [26]. Naderizadeh et al. demonstrated that beeswax-in-water emulsions could be used to produce hydrophobic or superhydrophobic coatings and that the resulting surface properties were influenced by wax-containing microstructures [24]. In a related beeswax emulgel system, Lombardo et al. used complementary microscopy methods to distinguish lipid domains from the surrounding stabilizing network [27]. Accordingly, the organization of the beeswax phase in the present composites is inferred from the SEM observations together with the increased contact angles and improved lubrication performance and was not directly mapped using a chemically selective technique.

### 3.4. FT-IR

**Figure 3:**
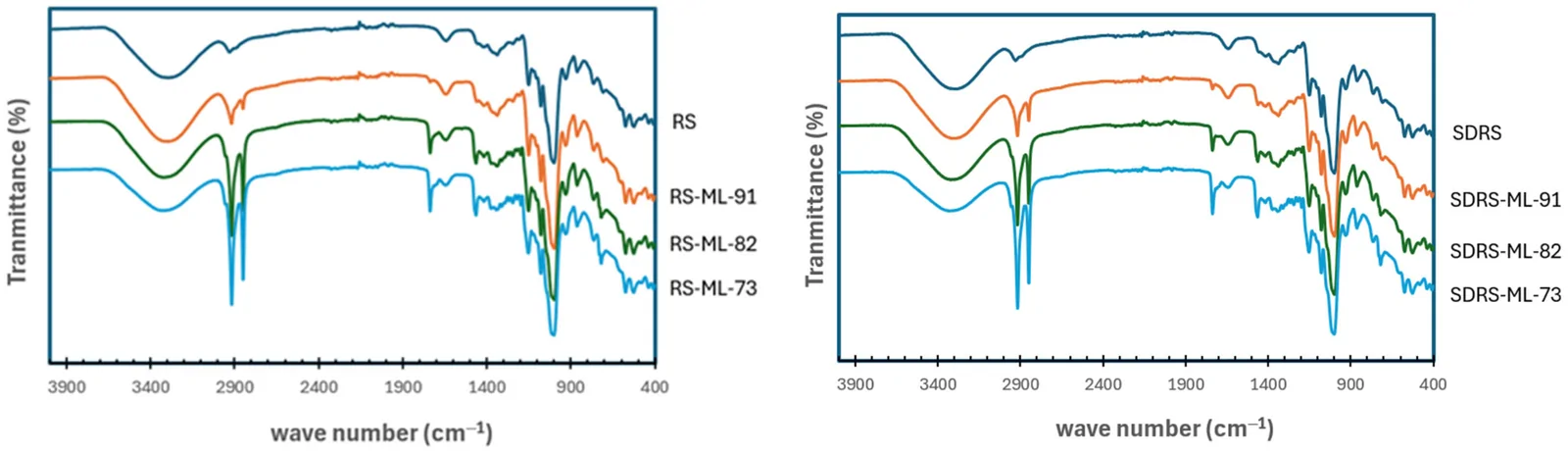
FT-IR spectra of native rice starch (RS) (**left**), spray-dried rice starch (SDRS) (**right**), and their beeswax composites at different starch-to-beeswax ratios (9:1, 8:2, and 7:3).

The FT-IR spectra of native rice starch (RS), spray-dried rice starch (SDRS), and their beeswax composites are shown in Figure 3. Both RS and SDRS exhibited the characteristic absorption bands of starch, including a broad O–H stretching band at approximately 3200–3400 cm^−1^, C–H stretching around 2920 cm^−1^, and prominent carbohydrate bands in the 1150–1000 cm^−1^ region corresponding to C–O and C–O–C vibrations. Following beeswax incorporation, the composite spectra retained the characteristic starch bands and showed increased contributions at approximately 2920 and 2850 cm^−1^, attributable to aliphatic C–H stretching, together with a band near 1730–1740 cm^−1^ associated with ester carbonyl groups in beeswax. Additional CH_2_ and CH_3_ vibrations were observed in the 1470–1370 cm^−1^ region. These assignments are consistent with previous FT-IR characterization of beeswax-containing materials, in which bands near 2915 and 2849 cm^−1^ were assigned to methylene stretching and the band near 1736 cm^−1^ to ester carbonyl stretching [24]. Comparable coexistence of polysaccharide- and beeswax-associated spectral features has also been reported in beeswax-modified starch-based composite materials [16]. These wax-associated spectral features were more evident at higher beeswax contents, indicating a greater contribution of the wax component to the composite spectra.

The FT-IR spectra of the RS- and SDRS-based composites were generally similar, indicating that the commercial spray-drying process did not produce major changes in the functional groups detectable by FT-IR. No new absorption bands or major peak shifts were observed after co-processing. This differs from chemically modified polysaccharide systems, in which the appearance of new carbonyl or carboxylate bands has been used as evidence of covalent derivatization [15]. The FT-IR results therefore confirm the coexistence of the characteristic chemical features of starch and beeswax within the analyzed composites and indicate that no detectable covalent modification occurred. Although non-covalent interactions are commonly reported in starch–lipid systems [28], FT-IR alone does not establish the spatial arrangement of the components or demonstrate a specific intermolecular interaction in the present composites. The proposed physical association between starch and beeswax is therefore inferred from the preparation process and the combined characterization findings rather than directly demonstrated by the spectra.

The increased contributions of aliphatic C–H and ester carbonyl bands are consistent with the presence of hydrophobic beeswax components in the composite samples. Beeswax is inherently hydrophobic, and its incorporation into starch-based materials has been reported to reduce surface wettability [15]. Together with the increased contact angles, the FT-IR findings are therefore consistent with reduced surface wettability after beeswax incorporation. However, FT-IR does not directly determine the distribution of beeswax at the particle surface and cannot by itself explain the measured contact-angle differences.

### 3.5. Tabletability of Co-Processed Starch-Beeswax Composites

**Figure 4:**
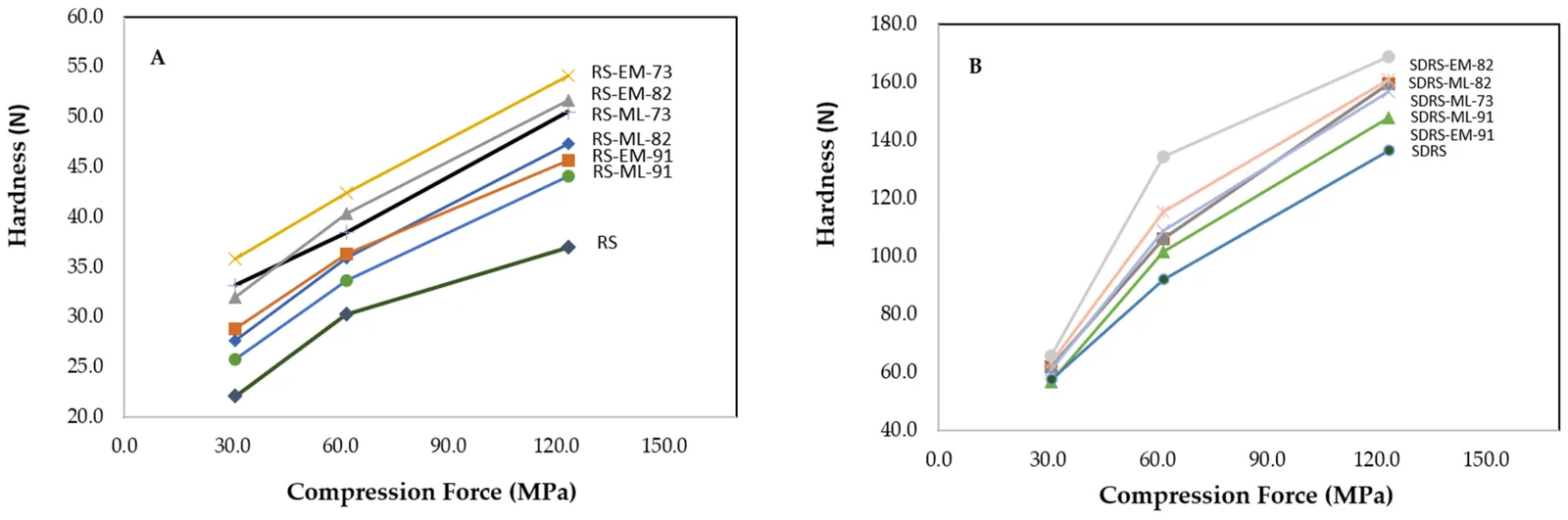
Tabletability profile of (**A**) co-processed rice starch/beeswax and (**B**) co-processed spray-dried rice starch/beeswax at different compression forces.

The tabletability profiles of the co-processed starch–beeswax powders are presented in Figure 4. Tablet hardness increased with compression force for all formulations. For the RS-based composites, tablet hardness increased from 25.7–35.7 N at 30.82 MPa to 44.0–54.1 N at 123.27 MPa, compared with native RS, which exhibited hardness values of 22.1, 30.3, and 36.9 N at 30.82, 61.64, and 123.27 MPa, respectively. The incorporation of beeswax markedly enhanced the compactibility of RS, particularly for the EM series. Among the RS composites, RS-EM-73 produced the highest hardness values throughout the compression range, reaching 54.1 N at 123.27 MPa.

In contrast, SDRS–beeswax composites exhibited considerably greater tabletability than their RS counterparts. Tablet hardness increased from 56.7–65.5 N at 30.82 MPa to 147.8–168.7 N at 123.27 MPa, whereas unmodified SDRS yielded hardness values of 57.4 and 136.3 N at the corresponding compression forces. The co-processed SDRS formulations showed pronounced improvements in hardness, particularly at intermediate and high compression forces. SDRS-EM-73 exhibited the highest tabletability among all formulations, producing hardness values of 65.5, 134.1, and 168.7 N at 30.82, 61.64, and 123.27 MPa, respectively.

Most co-processed formulations produced hardness values comparable to or greater than those of their corresponding starch bases, particularly at intermediate and high compression pressures, indicating that incorporation of beeswax did not compromise tabletability. The co-processed particles retained sufficient interparticulate bonding to generate mechanically robust tablets across the studied compression range.

SDRS-based formulations consistently produced higher hardness values than the corresponding RS-based formulations at all compression forces. This behavior can be attributed primarily to the inherent compaction properties of SDRS, which consists of spherical agglomerates formed during spray drying. These particles exhibit improved packing efficiency and facilitate deformation and interparticulate bond formation during compression, resulting in greater tablet strength than native rice starch [21,23].

The SEM observations showed that the characteristic spherical morphology of SDRS was largely preserved after co-processing and that no large, clearly separated secondary phase was visually apparent. Despite the marked increase in apparent surface hydrophobicity observed in the contact-angle measurements, tablet formation was not adversely affected. Although the spatial distribution and thickness of the beeswax phase were not directly determined, the preservation of tabletability suggests that beeswax incorporation did not substantially impair interparticulate bonding during compression.

A modest effect of the preparation method was also observed. In several formulations, emulsion-prepared composites produced slightly harder tablets than the corresponding melt-levigated systems, particularly in the SDRS series. This difference may reflect changes in particle aggregation or surface characteristics resulting from the emulsion-based process. However, the spatial distribution of beeswax was not directly determined, and a more homogeneous surface distribution cannot be concluded from the present results.

### 3.6. Compression Test and Tablet Evaluation

The compression characteristics and quality control parameters of tablets prepared with the tested lubricants are summarized in Table 2. All formulations were compressed using the preset pre-compression force (PCF) and main compression force (MCF) of 0.4 and 4.5 kN, respectively; the corresponding actual forces recorded during compression are reported in Table 2. Tablet weights were in the range between 295.4 ± 14.1 and 304.2 ± 11.2 mg with RSD less than 5%, indicating acceptable weight uniformity among all formulations. The incorporation of starch-beeswax composites as a lubricant at 2% concentration markedly reduced the ejection force compared with the control formulation without a lubricant (386.52 ± 36.95 N). RS-based composites lowered the ejection force to 146.20–339.88 N, whereas SDRS-based composites yielded ejection forces of 89.20–301.72 N. The higher ratio of beeswax in the composite resulted in the lower ejection force for both preparation methods. Among the co-processed lubricants, SDRS-EM-73 exhibited the lowest ejection force (89.20 ± 9.00 N), approaching that of the commercial lubricant HVO (0.5%) (93.04 ± 6.05 N), although magnesium stearate (0.5%) produced the lowest overall value (54.92 ± 3.59 N). Tablet hardness was also influenced by the lubricant type. The control tablets exhibited a hardness of 52.8 ± 4.3 N. RS-based composites produced hardness values between 50.0 ± 3.1 and 53.2 ± 3.7 N, while SDRS-based composites generated stronger tablets, ranging from 56.4 ± 3.9 to 60.9 ± 2.1 N. The highest hardness was observed for the SDRS-EM-73 formulation. The co-processed lubricants substantially improved tablet friability compared with the control formulation, which exhibited a friability of 12.02%. RS-based formulations showed friability values between 0.84% and 4.14%, whereas the values for SDRS-based formulations ranged from 0.16% to 1.57%. The lowest friability among the co-processed formulations was obtained with SDRS-EM-73 (0.16%), which was comparable to HVO (0.17%) but slightly higher than magnesium stearate (0.08%). Tablet disintegration times ranged from 35.35 to 45.87 s for the co-processed starch-beeswax formulations. These values were lower than that of magnesium stearate formulation (52.37 s) and comparable to that of HVO formulation (39.75 s), while remaining within acceptable limits for immediate-release tablets.

The compression and tablet evaluation results demonstrate that the lubrication performance of the co-processed starch–beeswax composites depended strongly on both beeswax content and particle structure. Although all co-processed materials exhibited lower ejection forces than the lubricant-free, control formulation (386.52 N), the extent of improvement varied considerably among formulations. Statistical analysis indicated that some formulations containing lower beeswax levels, particularly the 91-series composites, were not significantly different from the control or from one another despite showing numerically lower ejection force values. In contrast, formulations containing higher beeswax proportions generally produced significantly lower ejection forces, indicating enhanced lubrication efficiency. A progressive reduction in ejection force was observed as the beeswax content increased from the 91-series (10%) to the 73-series (30%) formulations, suggesting that the hydrophobic wax phase played a dominant role in reducing die-wall friction during tablet ejection. At comparable beeswax ratios, formulations containing SDRS-based composites consistently exhibited lower ejection forces than the corresponding RS-based formulations. The superior performance of the SDRS composites may be associated, at least in part, with the retention of their predominantly spherical agglomerated morphology after co-processing. This morphology may facilitate particle rearrangement and dispersion of the composite within the tablet blend. Together with the increased contact angles and the progressive decrease in ejection force with increasing beeswax content, these findings are consistent with a greater availability of the hydrophobic wax phase for reducing die-wall friction. However, neither the distribution of beeswax on the starch particles nor the formation of a continuous lubricating film was directly demonstrated. The most pronounced effect was observed for SDRS-EM-73, which produced the lowest ejection force among the co-processed lubricants (89.20 N), a value statistically comparable to that of HVO (93.04 N), indicating that this co-processed excipient achieved lubrication performance comparable to that of a commercially established lubricant.

Tablet weight was not significantly affected by lubricant type, indicating that all lubricants provided adequate powder flow and die-filling characteristics during tableting. Therefore, differences in tablet quality are primarily attributable to the influence of the lubricants on particle interactions during compression rather than variations in tablet mass. Tablet hardness is a sensitive indicator of lubricant-induced interference with interparticulate bonding, since lubricant surface coverage reduces particle–particle bonding and decreases tablet mechanical strength [29]. The significant differences observed among formulations demonstrate that lubricant composition markedly affected compactibility. Tablets containing starch–beeswax composites generally exhibited significantly greater hardness than those prepared with MGS and were comparable to or stronger than those containing HVO. These results suggest that the composites reduced the detrimental effects on tablet strength typically associated with hydrophobic lubricants such as MGS, which can interfere with interparticulate bond formation and consequently decrease tensile strength and increase brittleness, particularly at higher concentrations and longer blending times [30].

The friability results further support these observations. An effective lubricant should provide adequate die-wall lubrication to facilitate tablet ejection while minimizing disruption of interparticulate bonding and preserving tablet integrity [6,29]. Significant differences in friability were observed among formulations, with SDRS-based composites exhibiting substantially lower friability than most RS-based formulations. Although MGS and HVO produced the lowest friability values, several composite formulations, particularly SDRS-EM-82 and SDRS-EM-73, showed friability values statistically comparable to HVO and approached those of MGS. The combination of high hardness and low friability suggests that these composites provided effective lubrication while preserving mechanical strength, a key characteristic of an ideal lubricant [2,6].

Disintegration time is another critical quality attribute because excessive hydrophobicity can impede water penetration and delay tablet breakup [4,6]. Tablets prepared with starch–beeswax composites generally exhibited disintegration times comparable to or shorter than those containing HVO and consistently shorter than those prepared with MGS. The prolonged disintegration time of MGS-containing tablets suggests the formation of a hydrophobic surface film that retards water penetration and tablet disintegration [1,2,4,31,32]. In contrast, the incorporation of starch within the composite structure likely facilitated water uptake through capillary action and swelling upon hydration, thereby promoting disruption of the tablet matrix [33]. These effects may have partially counteracted the hydrophobic barrier imposed by beeswax [34], enabling relatively rapid tablet disintegration despite the increased tablet hardness.

These findings indicate that starch–beeswax composites, particularly SDRS-EM-73, can provide effective lubrication while maintaining acceptable tablet quality. However, only one batch of each starting material was evaluated, so batch-to-batch consistency and full reproducibility were not established. The purified beeswax was not supplied as a certified pharmacopoeial-grade excipient, and compliance of the specific rice starch batches with named pharmacopoeial monographs was not independently verified. In addition, these newly developed co-processed excipients lack dedicated pharmacopoeial monographs and standardized specifications. Their raw-material properties, composition, processing conditions, impurity profile, batch consistency, and functional performance therefore require further evaluation. Future studies should include multiple batches, different suppliers, and scale-up production to define critical material attributes and process parameters and to confirm the robustness and reproducibility of lubrication performance.

## 4. Conclusions

Starch–beeswax composites were successfully prepared from native rice starch and spray-dried rice starch using melt levigation and emulsification techniques. Incorporation of beeswax significantly increased surface hydrophobicity while preserving the fundamental physicochemical characteristics of the starch matrices. SEM demonstrated morphological and surface changes following co-processing, whereas FT-IR confirmed the coexistence of the characteristic spectral features of starch and beeswax without evidence of new covalent bond formation. Collectively, these findings are consistent with composite formation predominantly through physical incorporation of beeswax within the starch-based systems. The co-processed materials maintained acceptable flow properties and generally maintained or improved tabletability relative to the corresponding unmodified starches. SDRS-based composites consistently outperformed RS-based formulations, owing to the favorable spherical morphology and superior compactibility of spray-dried rice starch. The lubrication performance of the composites was strongly influenced by beeswax content and preparation method. Increasing beeswax concentration progressively reduced ejection force, while at matched starch-to-beeswax ratios, emulsion-prepared composites generally produced lower ejection forces than the corresponding melt-levigated composites. Among the formulations investigated, SDRS-EM-73 demonstrated the most desirable balance of properties, including high hydrophobicity, excellent tabletability, low ejection force, high tablet hardness, low friability, and acceptable disintegration time. SDRS-EM-73 reduced ejection force by approximately 77% compared with the lubricant-free control while maintaining tablet hardness and friability comparable to or better than those achieved with conventional lubricants. Further studies are required to establish standardized specifications, batch reproducibility, and scale-up feasibility before pharmaceutical application.

## Figures and Tables

**Table 1:** Moisture content, Carr’s index, Hausner ratio and water contact angle of co-processed rice starch/spray dried rice starch with beeswax at various ratios.

Sample Code	Base	BBR	MOP	MC (%)	CI (%)	HR	Contact Angle
RS-ML-91	RS	9:1	ML	10.57 ± 0.33	23.4 ± 1.0 ^cd^	1.31 ± 0.02 ^cd^	94.5 ± 1.5 ^h^
RS-ML-82	RS	8:2	ML	10.19 ± 0.41	25.1 ± 1.4 ^bc^	1.34 ± 0.03 ^bc^	110.6 ± 4.8 ^ef^
RS-ML-73	RS	7:3	ML	10.05 ± 0.29	27.0 ± 0.5 ^b^	1.37 ± 0.01 ^b^	118.2 ± 2.5 ^cd^
RS-EM-91	RS	9:1	EM	11.34 ± 0.75	22.6 ± 0.6 ^cd^	1.29 ± 0.01 ^cd^	95.9 ± 2.9 ^gh^
RS-EM-82	RS	8:2	EM	11.72 ± 0.66	24.6 ± 2.0 ^bc^	1.33 ± 0.10 ^bc^	114.7 ± 3.0 ^de^
RS-EM-73	RS	7:3	EM	11.14 ± 0.57	25.3 ± 1.6 ^bc^	1.34 ± 0.03 ^bc^	123.0 ± 1.6 ^bc^
SDRS-ML-91	SDRS	9:1	ML	9.78 ± 0.41	15.9 ± 0.6 ^f^	1.19 ± 0.01 ^f^	99.4 ± 2.3 ^g^
SDRS-ML-82	SDRS	8:2	ML	9.37 ± 0.38	19.2 ± 1.0 ^de^	1.24 ± 0.02 ^de^	116.4 ± 3.6 ^de^
SDRS-ML-73	SDRS	7:3	ML	9.52 ± 0.69	19.5 ± 1.4 ^de^	1.24 ± 0.02 ^de^	122.8 ± 5.7 ^bc^
SDRS-EM-91	SDRS	9:1	EM	10.12 ± 0.87	17.2 ± 1.9 ^ef^	1.21 ± 0.03 ^ef^	109.7 ± 4.5 ^f^
SDRS-EM-82	SDRS	8:2	EM	10.87 ± 1.02	24.3 ± 0.9 ^bc^	1.32 ± 0.02 ^bc^	118.9 ± 6.3 ^cd^
SDRS-EM-73	SDRS	7:3	EM	11.05 ± 1.04	26.2 ± 0.6 ^b^	1.35 ± 0.01 ^b^	125.1 ± 7.9 ^ab^
RS	-	-	-	10.23 ± 0.15	23.5 ± 1.3 ^cd^	1.31 ± 0.02 ^cd^	35.4 ± 2.0 ^j^
SDRS	-	-	-	8.97 ± 0.38	11.6 ± 1.4 ^g^	1.13 ± 0.02 ^g^	59.7 ± 2.7 ^i^
MGS	-	-	-	ND	33.3 ± 0.6 ^a^	1.50 ± 0.01 ^a^	133.6 ± 3.2 ^a^
HVO	-	-	-	ND	26.5 ± 0.4 ^b^	1.36 ± 0.01 ^b^	112.5 ± 5.3 ^ef^

BBR—Base-to-Beeswax ratio; CI (Carr’s index); EM—emulsion method; HR—Hausner’s ratio; HVO—hydrogenated vegetable oil; MC—moisture content; MGS—magnesium stearate; ML—melt levigation; MOP—method of preparation; ND—not determined, RS—rice starch, SDRS—spray-dried rice starch. Where applicable, different superscript letters within the same column indicate statistically significant differences (*p* < 0.05).

**Table 2:** Ejection force (N) and QC parameters of the tablet formulations containing various tested lubricants.

Formulation	Lubricant * (%)	PCF (kN)	MCF (kN)	Ejection Force (N)	Weight (mg)	Hardness (N)	Friability (%)	DT (s)
F-0	Control	0.4	4.7	386.52 ± 36.95 ^a^	303.1 ± 9.5	52.8 ± 4.3 ^b^	12.02	32.52
F-1	RS-ML-91—2%	0.4	4.7	339.88 ± 30.67 ^ab^	301.3 ± 9.8	50.0 ± 3.1 ^b^	4.14	39.68
F-2	RS-ML-82—2%	0.4	4.9	268.88 ± 19.10 ^e^	303.2 ± 7.2	51.0 ± 4.6 ^bc^	3.41	45.19
F-3	RS-ML-73—2%	0.4	4.8	187.44 ± 13.94 ^gh^	303.4 ± 6.5	52.1 ± 4.7 ^bc^	0.89	44.95
F-4	RS-EM-91—2%	0.4	4.6	323.76 ± 31.68 ^bc^	299.5 ± 6.1	51.2 ± 3.6 ^bc^	3.39	35.35
F-5	RS-EM-82—2%	0.4	4.8	241.96 ± 24.58 ^f^	299.0 ± 10.7	52.6 ± 2.2 ^bc^	1.89	40.30
F-6	RS-EM-73—2%	0.4	4.9	146.20 ± 10.78 ^i^	295.4 ± 14.1	53.2 ± 3.7 ^bc^	0.84	44.65
F-7	SDRS-ML-91—2%	0.4	4.7	301.72 ± 20.16 ^cd^	299.1 ± 9.1	56.4 ± 3.9 ^cd^	1.57	40.39
F-8	SDRS-ML-82—2%	0.4	4.6	194.76 ± 16.43 ^gh^	299.6 ± 11.6	57.9 ± 3.0 ^de^	0.61	39.50
F-9	SDRS-ML-73—2%	0.4	4.6	111.76 ± 11.20 ^jk^	304.2 ± 11.2	60.1 ± 3.2 ^f^	0.35	40.50
F-10	SDRS-EM-91—2%	0.4	4.7	288.36 ± 31.41 ^de^	299.5 ± 12.1	57.4 ± 2.4 ^de^	0.89	45.87
F-11	SDRS-EM-82—2%	0.4	4.7	168.00 ± 16.23 ^h^	300.8 ± 13.1	59.4 ± 3.1 ^ef^	0.23	42.50
F-12	SDRS-EM-73—2%	0.4	4.6	89.20 ± 9.00 ^k^	302.5 ± 7.3	60.9 ± 2.1 ^f^	0.16	44.80
F-MGS	MGS—0.5%	0.4	4.6	54.92 ± 3.59 ^L^	299.1 ± 10.5	40.2 ± 3.3 ^a^	0.08	52.37
F-HVO	HVO—0.5%	0.4	4.7	93.04 ± 6.05 ^jk^	300.2 ± 11.2	54.4 ± 4.3 ^bcd^	0.17	39.75

* Control—no lubricant; DT—disintegration time; EM—emulsified beeswax; HVO—hydrogenated vegetable oil (Lubritab); MCF—main compression force; MGS—magnesium stearate; ML—melt levigation; PCF—pre-compression force; RS—rice starch; SDRS—spray dried rice starch. Ejection force, tablet weight, and hardness values are expressed as mean ± standard deviation. Where applicable, different superscript letters within the same column indicate statistically significant differences (*p* < 0.05).

## Data Availability

The original contributions presented in this study are included in the article. Further inquiries can be directed to the corresponding author.
